# Associations of Intracranial Hemorrhage and Extracranial Injury with Outcomes in Patients with Traumatic Brain Injury

**DOI:** 10.7759/cureus.93414

**Published:** 2025-09-28

**Authors:** Gregory S Huang, C. Michael Dunham

**Affiliations:** 1 Trauma, Critical Care, and General Surgery Services, St. Elizabeth Youngstown Hospital, Youngstown, USA

**Keywords:** admission glucose, adverse outcome, brain ct scan, extracranial injury, glascow coma scale, injury severity score, intracranial hemorrhage, trauma mortality, traumatic brain injury

## Abstract

Background

Researchers have shown that extracranial (EC) injuries can influence outcomes in patients with intracranial hemorrhage (ICH). Large data sets have noted that the presence of EC injury can have detrimental effects on ICH. However, the effects are not well quantified. EC injury has been historically treated as a categorical variable. Only one study besides the current one has attempted to calculate the degree of EC injury in affecting outcomes in ICH. We calculated a novel systemic variable for EC injury as the EC Injury Severity Score (EC-ISS) and aimed to assess the impact of EC injuries on ICH patient outcomes in blunt trauma.

Objective

Our primary objective was to examine the impact of EC injury on six undesirable outcomes in blunt ICH trauma: (1) mortality, (2) ICU stay ≥2 days, (3) hospital stay >5 days, (4) adverse outcome (AO; mortality, ICU stay ≥2 days, or hospital stay >5 days), (5) no commands at hospital discharge, and (6) no commands at three months. Secondary objectives included comparing EC-ISS and ICH Abbreviated Injury Scale scores (AIS) with ISS.

Methods

This was a retrospective analysis of adult trauma patients with ICH admitted to a level I trauma center in northeast Ohio from January 21 to July 21 during each year in 2018, 2019, and 2020 (the parent data set was obtained from a prior COVID-19 publication). The population had computed tomography (CT)-confirmed ICH, admission Glasgow Coma Scale (GCS) score of 3-15, and blunt trauma. CT ICH mass effect scores were rated as one each for lateral ventricular compression, basal cistern compression, and midline shift (0-3). The ICH CT score was mass effect score plus subarachnoid hemorrhage (SAH; 0-5). ICH AIS, ISS, and admission hypotension (systolic blood pressure <100 mmHg) were from the trauma registry (TR). EC-ISS was ISS minus the ICH AIS score squared. GCS deficit was 15 minus GCS. AOs were hospital death, ICU stay ≥2 days, or hospital stay >5 days. Admission glucose was from the electronic medical record.

Results

The study included 436 patients. Hospital death was independently associated with ICH mass effect (p<0.0001), age (p=0.0002), EC-ISS (p=0.0002), and ICH AIS (p=0.0010) but not with ISS ≥16 (p=0.8243). Intensive care days had independent associations with GCS (p<0.0001), admission glucose (p = 0.0003), CT score (p<0.0001), EC-ISS (p<0.0001), ICH AIS (p<0.0001), and hypotension (p = 0.0493), but not with ISS ≥16 (p = 0.5517). Hospital days had independent associations with GCS (p<0.0001), admission glucose (p<0.0001), CT score (p=0.0007), EC-ISS (p<0.0001), and ICH AIS (p<0.0001), but not with ISS ≥16 (p=0.2359). AO had independent associations with GCS (p<0.0001), admission glucose (p<0.0016), EC-ISS (p=0.0102), ICH AIS (p<0.0001), hypotension (p=0.0347), and CT score (p=0.0215), but not with ISS ≥16 (p=0.2011). Not following commands at discharge had independent associations with GCS (p=0.0006), EC-ISS (p=0.0459), ICH AIS (p=0.0002), and CT score (p=0.0201), but not with ISS ≥16 (p=0.9500). Not following commands at three months had independent associations with EC-ISS (p<0.0001), ICH AIS (p=0.0006), age (p<0.0001), GCS deficit + EC-ISS (p<0.0001), and hypotension (p=0.0078), but not with ISS ≥16 (p=0.4157).

Conclusion

ICH AIS and EC-ISS have independent associations with six undesirable ICH outcomes. ICH AIS and EC-ISS may better represent risk conditions in ICH patients when compared with ISS.

## Introduction

Traumatic brain injury (TBI) is a leading cause of death and disability worldwide [1]. Numerous studies have examined risk conditions in intracranial head injury (ICH) patients with a blunt trauma mechanism. 

Scoring methods such as the Glasgow Coma Scale (GCS) have been associated with poor outcomes. Early postinjury GCS has been associated with in-hospital mortality and lower posthospital discharge GCS values [2,3]. Increased CT head scores, such as the Marshall categorical system and Rotterdam CT score, which delineate ICH mass effect, have demonstrated associations with in-hospital mortality [4]. Another investigation showed that the mass effect score was a better indicator for the need for surgical decompression when compared with GCS [5]. 

Systemic predictors such as Injury Severity Score (ISS) and ICH Abbreviated Injury Score (AIS) have also been well studied in this population. There is evidence that ISS alone has limitations in predicting outcomes. Recent publications have shown that greater ISS, when combined with decreased GCS and advanced age, is a more accurate predictor [6, 7] than ISS alone. Multiple investigators have shown that ICH AIS scores are associated with increased ICU length of stay (LOS) and hospital mortality [8] as well as 12-month unfavorable Glasgow Outcome Scale scores [9].

Hypotension on arrival at the trauma center is another well-established risk factor for increased mortality [10]. A recent meta-analysis with over 400,000 patients showed a two-fold odds of mortality with hypotension and TBI [11]. Hyperglycemia on admission has been shown to be a risk condition for mortality and undesirable outcomes in blunt trauma TBI patients [12,13].

Collectively, these risk factors have been shown in the literature to affect ICH outcomes unfavorably. However, the effects of extracranial (EC) injury on the outcome of TBI have not been well quantified in the literature. Most research has treated EC injury as a categorical value. An older meta-analysis from 2012 that included 39,000 patients concluded that major EC injury was an important factor for mortality in TBI patients [14]. The Corticosteroid Randomisation After Significant Head Injury (CRASH) trial collaborators developed a simple model that noted the presence of major EC injury as a prognostic factor for TBI [15].

We have found only one study that treated EC injury as a systemic variable by calculating EC-ISS values in patients with TBI. Skrifvars’s post hoc study on the administration of erythropoietin versus placebo provides results comparing patients with EC-ISS >6 versus EC-ISS ≤6 [16]. ICU survival, hospital survival, six-month survival, and six-month good outcome proportions were similar for the two groups. However, ICU and hospital LOS were significantly increased with EC-ISS >6. Therefore, their model did not discriminate for the outcome.

Because of the lack of research on EC-ISS as a severity metric in head injury, we calculated a novel EC-ISS that included the same components of the ISS, excluding head components. EC-ISS was ISS minus the ICH AIS score squared. 

Our primary objective was to assess the impact of EC injuries on ICH patient outcomes in blunt trauma. Specifically, six undesirable outcomes were examined: (1) mortality, (2) ICU stay ≥2 days, (3) hospital stay >5 days, (4) adverse outcome (AO; mortality, ICU stay ≥2 days, or hospital stay >5 days), (5) no commands at hospital discharge, and (6) no commands at three months. Secondary objectives included comparing EC-ISS and ICH AIS with ISS.

## Materials and methods

Ethics statements

The current retrospective study was reviewed by the local institutional review board (IRB) of Bon Secours Mercy Health, Cincinnati, OH, and determined to be qualified for exemption according to Exempt Category 4 (IRB number: 2025-Trauma-Huang). The need for informed consent was waived due to minimal risk, the use of a de-identified registry, and the retrospective nature of the investigation. 

Study design and population

The parent group data source was adult patients admitted to a level I trauma center in Northeast Ohio from January 21 to July 21 during each year 2018, 2019, and 2020, as described in a previous COVID-19 publication [17] that addressed the Ohio stay-at-home order. Of the total 2,076 trauma patients, 1,045 had either trauma team (high-level acuity) or trauma alert (mid-level acuity) activation, and 1,031 had trauma consultation (non-activation).

Inclusion and exclusion criteria

Included patients were 18 years or older with blunt trauma ICH, were triaged as a trauma team, alert, or consult (non-activation), and were admitted to the hospital. Trauma transfers that were admitted to the trauma service were also included. Patients were excluded if they were younger than 18 years, had been discharged from the emergency department, or had penetrating trauma. There was no missing data on the included patients. 

Data collection

Data obtained from the trauma registry (TR) included mechanism of injury, age, GCS, ISS, blood pressure, hospital transfer or scene designation, hospital death, ICU stay (days), hospital stay (days), and activation or consultation status. Activation patients were those with full trauma team activation or trauma alert (partial trauma team) activation, whereas consultation patients received no activation process. Information from the EPIC electronic medical records (EMR) included trauma activation and consultation patients, and the presence or absence of ICH. These inquiries were pursued sequentially in each trauma patient to determine the study results. The presence or absence of ICH in activation and consultation patients was determined by a method described previously [5]. 

The study group was created, which consisted of the activation and consultation of patients with an ICH and a blunt trauma mechanism. For the study group, we obtained from the TR a designation of those without or with subarachnoid hemorrhage (SAH) and the highest head AIS scores. An extracranial ISS value was calculated from the total ISS and the ICH AIS values. EC-ISS = Total ISS (minus) ICH AIS SQUARED (ICH AIS x ICH AIS). This was done to avoid overestimation of EC injury burden. There is only one other study that calculated EC-ISS [16], since there is no conventional standard.

The following data were obtained via EMR audits: ICH mass effect score, ICH CT score, inability to follow commands at hospital discharge (yes or no), inability to follow commands at three months postinjury (yes or no), and admission serum glucose. GCS deficit was calculated as 15 minus GCS. 

Not following commands at the trauma center, discharge was obtained from the EMR discharge summary and pre-discharge progress notes. The documentation of the following commands at three months was obtained from the EMR, which included hospital readmissions, office visits, and follow-up appointments.

The ICH mass effect score was described in the hypertonic saline investigation [18] and used to quantify the ICH mass effect. The ICH mass effect score was calculated as the sum of midline shift, lateral ventricle compression, and basal cistern compression. Each of the three findings was given a value of 0 if absent and 1 if present (theoretical range, 0-3).

**Figure 1:**
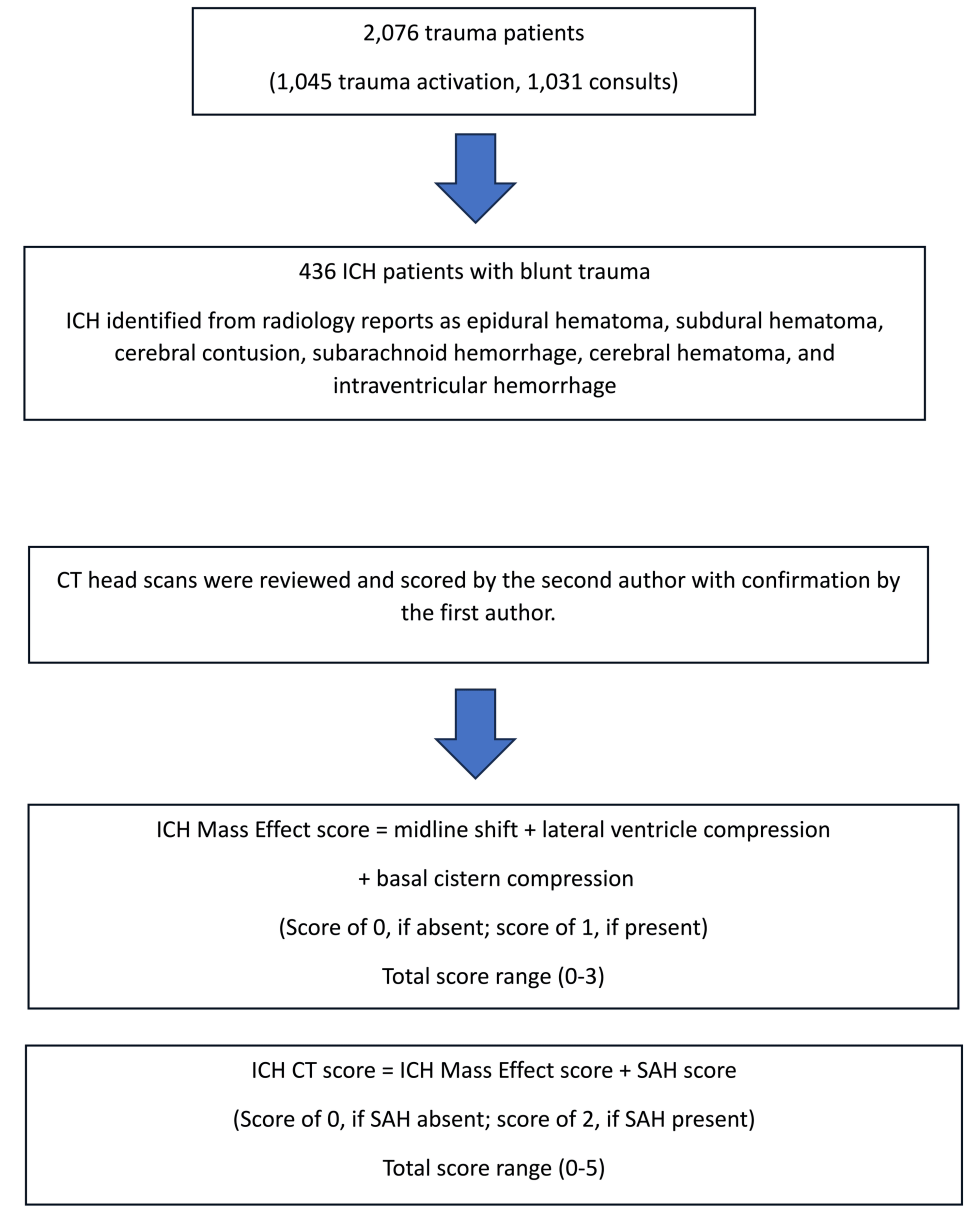
ICH mass effect score and ICH CT score ICH: intracranial hemorrhage; CT: computed tomography; SAH: subarachnoid hemorrhage

The ICH CT score was described in a prior publication and was calculated by taking the ICH mass effect score and adding an SAH component [5]. When SAH was present, the value was 2, whereas if it was absent, the value was 0. The range of the ICH CT score was 0-5. The second author reviewed all axial and coronal slices of each patient’s CT scan in order to calculate the ICH mass effect and ICH CT score. Inter-observer reliability was confirmed by the first author. When scoring was different, the two authors reviewed each image together in order to obtain a consensus (Figure 1).

Deaths were the sum of the hospital deaths and patients discharged to hospice. Other investigators of trauma patients with ICH have considered discharge to hospice to be a fatal, terminal event [19]; therefore, combining the deaths is appropriate.

AO is a specific undesirable outcome defined as hospital mortality, ICU stay ≥2 days, or hospital stay >5 days.

We have published other investigations using this definition of AO (mortality, ICU LOS ≥ 2 days, or hospital LOS > 5 days) [6, 7].

There are six undesirable outcomes: (1) mortality, (2) ICU stay ≥2 days, (3) hospital stay >5 days, (4) AO, (5) no commands at hospital discharge, and (6) no commands at three months.

Statistical analysis

All analyses included the 436 patients from the study group. There were no imputations because no data were missing. Continuous data are presented as the mean and standard deviation, proportional data as the count (N) and percent, and ordinal data as the median and a percentile range. 

T-tests were performed to assess differences between two groups of continuous data (age and admission glucose). The assumption for t-tests was normality, and the Wilcoxon test was used for non-normal data. For dichotomous proportional data conforming to a 2 x 2 contingency table format (GCS 3-12, ICH mass effect, ICH CT score ≥3, ISS ≥16, ICH AIS ≥4, EC-ISS ≥6, systolic blood pressure <100 mmHg, and SAH), the two-tailed Fisher's exact test was employed. The chi-square-p, odds ratio (OR), and risk ratio (RR) were computed to quantify proportional differences. For nonparametric data (EC-ISS), the median and a percentile range are presented. When comparing the median between two groups, the Wilcoxon rank sum-p was tested. With nonparametric testing, SAS (SAS Inc., Cary, NC, USA) routinely computes an analysis of variance (t-test for two groups) to provide a comprehensive baseline analysis of group differences and a comparison with nonparametric results.

Multivariate regression analysis was used to assess the simultaneous associations of all independent variables with a dependent variable. Multivariate logistic regression was used for data with (p<0.10) dichotomous dependent variables (mortality, AO, no hospital discharge commands, and no commands at three months). Multivariate linear regression was used for data with a continuous dependent variable (ICU LOS and hospital LOS).

Data were entered into Excel 2010 (Microsoft Corp., Redmond, WA, USA) and imported into SAS System for Windows (release 9.2). The significance level for the p-value was set at <0.05.

## Results

The patient characteristics are shown in Table 1. The mean glucose was 140 mg/dL. 136 out of 436 patients had an admission glucose concentration ≥150 mg/dL: 46 (33.8%) had a history of diabetes mellitus; 90 (66.2%) did not have a diagnosis of diabetes mellitus. The death rate was low; out of the 29 deaths, three were hospice discharges. The correlation of ICH AIS and ISS was P<0.0001; r=0.72; r²=0.51; ICH AIS accounted for only 50% of the variation in total ISS, suggesting that EC injuries are often present.

**Table 1:** Traits and outcomes of patients in the current study EC-ISS, Extracranial ISS = Total ISS (minus) ICH AIS SQUARED (ICH AIS x ICH AIS)

Variable	
Total patients	436
Male (N, %)	222 (50.9%)
Age (years) (mean, SD) (range)	68.5±19.8 (18–100)
Glasgow Coma Scale (GCS) Score 3–12 (N, %)	64 (14.7%)
GCS Score 13–14 (N, %)	88 (20.2%)
GCS Score 15 (N, %)	284 (65.1%)
Intracranial hemorrhage (ICH) mass effect (N, %)	35 (8.0%)
ICH CT score 0–1 (N, %)	184 (42.2%)
ICH CT score 2 (N, %)	220 (50.5%)
ICH CT score 3–5 (N, %)	32 (7.3%)
Subdural hemorrhage (SAH) (N, %)	242 (55.5%)
Injury Severity Score (ISS) 16–75 (N, %)	241 (55.3%)
ICH Abbreviated Injury Scale (AIS) 1–2 (N, %)	113 (25.9%)
ICH AIS 3 (N, %)	102 (23.4%)
ICH AIS 4–5 (N, %)	221 (50.7%)
Extracranial (EC)-ISS 0–5 (N, %)	368 (84.4%)
EC-ISS 6–71 (N, %)	68 (15.6%)
EC-ISS 6–71 median (25%–75%)	11.5 (9.0–18.0)
Glucose (mean, SD) (range)	140.3±52.1 (61–442)
Systolic blood pressure (sBP) <100 (N, %)	19 (4.4%)
Hospital death (N, %)	29 (6.7%)
ICU length of stay (LOS) ≥2 d (N, %)	138 (31.7%)
Hospital LOS >5 d (N, %)	160 (36.7%)
Adverse outcome (N, %)	196 (45.0%)
No discharge commands (N, %)	44 (10.1%)
No three-month commands (N, %)	35 (8.0%)

Univariate associations with in-hospital mortality are presented in Table 2. Death was associated with GCS 3-12, ICH mass effect, ICH CT score ≥3, ISS ≥16, ICH AIS ≥4, and sBP <100. The associations (OR and RR) were large. Age was increased in the hospital death group. Although EC-ISS ≥6 was not significantly associated with death, the OR and RR were greater with death. 

**Table 2:** Univariate risk condition associations with hospital mortality χ2: chi-square value; OR: odds ratio; RR: risk ratio;╠t Value=-1.9

Variable	Lived	Died	T-test-p	χ^2^	χ^2^-p	OR	RR	Wilcoxon-p
Total	407 (93.3%)	29 (6.7%)	–	–	–	–	–	–
Glasgow Coma Scale (GCS) 3–12 (N, %)	48 (11.8%)	16 (55.2%)	–	40.7	<0.0001	9.2	4.7	–
Intracranial hemorrhage (ICH) mass effect (N, %)	22 (5.4%)	13 (44.8%)	–	57.0	<0.0001	14.2	8.3	–
ICH CT score ≥3 (N, %)	20 (4.9%)	12 (43.4%)	–	52.9	<0.0001	13.7	8.4	–
Injury Severity Score (ISS) ≥16 (N, %)	214 (52.6%)	27 (93.1%)	–	18.0	<0.0001	12.2	1.8	–
ICH Abbreviated Injury Scale scores (AIS) ≥4 (N, %)	195 (47.9%)	26 (89.7%)	–	18.9	<0.0001	9.4	1.9	–
Extracranial (EC)-ISS ≥6 (N, %)	62 (15.2%)	6 (20.7%)	–	0.6	0.4340	1.5	1.4	–
EC-ISS median (25%–90%)	1 (0–9)	1 (0–25)	–	–	–	–	–	0.1255
Age (mean, SD)	68.0±19.7	75.3±19.4	0.0563^╠^	–	–	–	–	–
Blood pressure <100	15 (3.7%)	4 (13.8%)	–	6.6	0.0100	4.2	3.7	–

Multivariate logistic regression analysis showed in-hospital death was independently associated with increased ICH mass effect (p<0.0001), increased age (p=0.0002), increased EC-ISS (p=0.0002), and increased ICH AIS (p=0.0010), but not with ISS ≥16 (p=0.8243) or GCS 3-12 (p=0.1568). The R-squared value was 0.22.

Univariate associations with ICU days are presented in Table 3. ICU days were associated with GCS 3-12, ICH mass effect, ICH CT score ≥ 3, ISS ≥16, EC-ISS ≥6, ICH AIS ≥4, SAH, and sBP <100. OR was large for all these variables, except for SAH. Admission glucose was increased in the group with longer ICU days.

**Table 3:** Univariate risk condition associations with increased ICU stay χ2: chi-square value; OR: odds ratio; RR: risk ratio; ╠t Value=-4.3

Variable	0–1 Days	≥2 Days	T-test-p	χ^2^	χ^2^-p	OR	RR
Total	298 (68.3%)	138 (31.7%)	–	–	–	–	–
Glasgow Coma Scale (GCS) score 3–12 (N, %)	8 (2.7%)	56 (40.6%)	–	108.1	<0.0001	24.8	15.1
Intracranial hemorrhage (ICH) mass effect (N, %)	4 (1.3%)	31 (22.5%)	–	57.0	<0.0001	21.3	16.8
ICH CT score ≥3 (N, %)	1 (0.3%)	31 (22.5%)	–	67.9	<0.0001	96.1	74.9
Injury Severity Score (ISS) ≥16 (N, %)	124 (41.6%)	117 (84.8%)	–	71.1	<0.0001	7.8	2.0
ICH Abbreviated Injury Scale scores (AIS)≥4 (N, %)	115 (38.6%)	106 (76.8%)	–	55.1	<0.0001	5.3	2.0
Extracranial (EC)-ISS ≥6 (N, %)	26 (8.7%)	42 (30.4%)	–	33.8	<0.0001	4.6	3.5
Glucose (mean, SD)	132.6±48.1	156.7±56.6	<0.0001^╠^	–	–	–	–
Subarachnoid hemorrhage (SAH) (N, %)	155 (52.0%)	87 (63.0%)	–	4.6	0.0311	1.6	1.2
Systolic blood pressure (sBP) <100 (N, %)	7 (2.4%)	12 (8.7%)	–	9.1	0.0025	4.0	3.7

Multivariate linear regression analysis showed increased ICU days was associated with decreased GCS (p<0.0001), increased glucose (p = 0.0003), increased ICH CT score (p<0.0001), increased EC-ISS (p<0.0001), increased ICH AIS (p<0.0001) and systolic blood pressure <100 (p = 0.0493), but not with ISS ≥16 (p=0.5517). The R-squared value was 0.55. 

Univariate associations with hospital days are presented in Table 4. Hospital days were associated with GCS 3-12, ICH mass effect, ICH CT Score ≥ 3, ISS ≥16, EC-ISS ≥6, ICH AIS ≥4, and SAH. The OR was larger, except for SAH. Elevated glucose levels on presentation were associated with longer hospital days.

**Table 4:** Univariate risk condition associations with increased hospital stay χ2: chi-square value; OR: odds ratio; RR: risk ratio; ╠t Value=-4.8

Variable	1–5 Days	>5 Days	T-test-p	χ^2^	χ^2^-p	OR	RR
Total	276 (63.3%)	160 (36.7%)	–	–	–	–	–
Glasgow Coma Scale (GCS) 3–12 (N, %)	13(4.7%)	51 (31.9%)	–	59.7	<0.0001	9.5	6.8
Intracranial hemorrhage (ICH) mass effect (N, %)	6 (2.2%)	29 (18.1%)	–	34.9	<0.0001	10.0	8.4
ICH CT Score ≥3 (N, %)	4 (1.5%)	28 (17.5%)	–	38.4	<0.0001	14.4	12.1
Injury Severity Scores (ISS) ≥16 (N, %)	113 (40.9%)	128 (80.0%)	–	62.5	<0.0001	5.8	2.0
ICH Abbreviated Injury Scale scores (AIS) ≥4 (N, %)	107 (38.8%)	114 (72.3%)	–	42.8	<0.0001	3.9	1.8
Extracranial (EC)-ISS ≥6 (N, %)	23 (8.3%)	45 (28.1%)	–	30.1	<0.0001	4.3	3.4
Glucose (mean, SD)	130.6±42.6	157.0±62.0	<0.0001^╠^	–	–	–	–
Subarachnoid hemorrhage (SAH) (N, %)	141 (51.0%)	101 (63.1%)	–	5.9	0.0148	1.6	1.2

Multivariate linear regression analysis showed increased hospital days were associated with decreased GCS (p<0.0001), increased glucose (p<0.0001), increased ICH CT score (p=0.0007), increased EC-ISS (p<0.0001), and increased ICH AIS (p<0.0001), but not with ISS ≥16 (p=0.2359). The R-squared value was 0.45.

Univariate associations with AO are presented in Table 5. AO was associated with GCS 3-12, ICH mass effect, ICH CT score ≥ 3, ISS ≥ 16, EC-ISS ≥ 6, ICH AIS ≥ 4, and sBP < 100. OR was large for all these variables. Elevated glucose levels were moderately associated with AO.

**Table 5:** Univariate risk condition associations with AO AO: adverse outcome;  χ2: chi-square value; OR: odds ratio; RR: risk ratio; ╠t Value=-5.3

Variable	AO: No	AO: Yes	T-test-p	χ^2^	χ^2^-p	OR	RR
Total	240 (55.0%)	196 (45.0%)	–	–	–	–	–
Glasgow Coma Scale (GCS) 3–12 (N, %)	3 (1.3%)	61 (31.1%)	–	76.9	<0.0001	35.7	24.9
Intracranial hemorrhage (ICH) mass effect (N, %)	1 (0.4%)	34 (17.4%)	–	41.9	<0.0001	50.2	41.3
ICH CT Score ≥3 (N, %)	0 (0%)	32 (16.3%)	–	42.3	<0.0001	–	–
Injury Severity Scores (ISS) ≥16 (N, %)	86 (35.8%)	155 (79.1%)	–	81.6	<0.0001	6.8	2.2
ICH Abbreviated Injury Scale scores (AIS) ≥4 (N, %)	80 (33.3%)	141 (71.9%)	–	64.3	<0.0001	5.1	2.2
Extracranial (EC)-ISS ≥6 (N, %)	19 (7.9%)	49 (25.0%)	–	23.9	<0.0001	3.9	3.2
Glucose (mean, SD)	128.3±41.9	154.9±59.2	<0.0001^╠^	–	–	–	–
Systolic blood pressure (sBP) <100 (N, %)	4 (1.7%)	15 (7.7%)	–	9.3	0.0023	4.9	4.6

Multivariate logistic regression analysis of the study group demonstrated AO had independent associations with decreased GCS (p<0.0001), increased glucose (p<0.0016), increased EC-ISS (p=0.0102), increased ICH AIS (p<0.0001), systolic blood pressure <100 (p=0.0347), and increased ICH CT score (p=0.0215), but not with ISS ≥16 (p=0.2011). The R-squared value was 0.31.

Univariate associations with not following commands at discharge are presented in Table 6. Not following commands at discharge was associated with GCS 3-12, ICH mass effect, ICH CT score ≥ 3, ISS ≥ 16, EC-ISS ≥ 6, and ICH AIS ≥ 4. All the odds ratios were moderate to large. Elevated glucose was statistically associated with not following commands at discharge.

**Table 6:** Univariate risk condition associations with not following commands at hospital discharge FCs: following commands at discharge; χ2: chi-square value; OR: odds ratio; RR: risk ratio; ╠t Value=-2.0

Variable	FCs: Yes	FCs: No	T-test-p	χ^2^	χ^2^-p	OR	RR
Total	392 (89.9%)	44 (10.1%)	–	–	–	–	–
Glasgow Coma Scale (GCS) 3–12 (N, %)	33 (8.4%)	31 (70.5%)	–	121.6	<0.0001	25.9	8.4
Intracranial hemorrhage (ICH) mass effect (N, %)	15 (3.8%)	20 (45.5%)	–	92.9	<0.0001	20.9	11.9
ICH CT Score ≥3 (N, %)	13 (3.3%)	19 (43.2%)	–	92.4	<0.0001	22.2	13.0
Injury Severity Scores (ISS) ≥16 (N, %)	200 (51.0%)	41 (93.2%)	–	28.5	<0.0001	13.1	1.8
ICH Abbreviated Injury Scale scores (AIS) ≥4 (N, %)	182 (46.4%)	39 (88.6%)	–	28.2	<0.0001	9.0	1.9
Extracranial (EC)-ISS ≥6 (N, %)	53 (13.5%)	15 (34.1%)	–	12.7	0.0004	3.3	2.5
Glucose (mean, SD)	138.6±52.1	155.2±49.9	0.0450^╠^	–	–	–	–

Multivariate logistic regression demonstrated that not following commands at discharge had independent associations with decreased GCS (p=0.0006), increased EC-ISS (p=0.0459), increased ICH AIS (p=0.0002), and increased ICH CT score (p=0.0201), but not with ISS ≥16 (p=0.9500). The R-squared value was 0.34.

Univariate associations with not following commands at three months are presented in Table 7. Not following commands at three months was associated with GCS 3-12, ICH mass effect, ICH CT score ≥ 3, ISS ≥ 16, ICH AIS ≥ 4, and sBP < 100. All variables had large ORs. The GCS deficit and GCS deficit + EC-ISS scores were higher when not following commands at 3 months. For those not following commands at three months, the GCS deficit + EC-ISS scores were significantly greater when compared with the GCS deficit scores (Wilcoxon p<0.0001). Older age was associated with not following commands at three months.

**Table 7:** Univariate risk condition associations with not following commands at three months FC: following commands at three months; χ2: chi-square value; OR: odds ratio; RR: risk ratio; ╠t Value=-2.1

Variable	FCs: Yes	FCs: No	T-test-p	χ^2^	χ^2^-p	OR	RR	Wilcoxon-p
Total	401 (92.0%)	35 (8.0%)	–	–	–	–	–	–
Glasgow Coma Scale (GCS) 3–12 (N, %)	44 (11.0%)	20 (57.1%)	–	54.8	<0.0001	10.8	5.2	–
Intracranial hemorrhage (ICH) mass effect (N, %)	21 (5.2%)	14 (40.0%)	–	52.7	<0.0001	12.1	7.6	–
ICH CT score ≥3 (N, %)	19 (4.7%)	13 (37.1%)	–	49.7	<0.0001	11.9	7.8	–
Injury Severity Scores (ISS) ≥16 (N, %)	200 (51.0%)	41 (93.2%)	–	28.5	<0.0001	13.1	1.8	–
ICH Abbreviated Injury Scale scores (AIS) ≥4 (N, %)	190 (47.4%)	31 (88.6%)	–	21.9	<0.0001	8.6	1.9	–
GCS deficit median (IQR)	0 (0–1)	4 (0–9)	–	–	–	–	–	<0.0001
GCS deficit + Extracranial (EC)-ISS Score median (IQR)	1 (1–5)	8 (1–12)	–	–	–	–	–	0.0006
Age (mean, SD)	67.9±19.8	75.2±19.1	0.0359^╠^	–	–	–	–	–
Systolic blood pressure (sBP) <100 (N, %)	14 (3.5%)	5 (14.3%)	–	9.0	0.0027	4.6	4.1	–

Multivariate logistic regression demonstrated that not following commands at three months had independent associations with increased EC-ISS (p<0.0001), increased ICH AIS (p=0.0006), increased age (p<0.0001), increased GCS deficit + EC-ISS score (p<0.0001), and sBP <100 (p=0.0078), but not with ISS ≥16 (p=0.4157) or GCS 3-12 (p=0.4248). The R-squared value was 0.22.

Table 8 is a display of the multivariate regression analysis summary results for the six undesirable outcome models. EC-ISS and ICH AIS were independent associations for each model outcome, but ISS ≥16 was not.

**Table 8:** Multivariate regression analysis summary results for undesirable outcome models EC-ISS: extracranial-Injury Severity Score; ICH AIS: intracranial hemorrhage Abbreviated Injury Scale scores; ISS: Injury Severity Scores

Model	EC-ISS p-values	ICH AIS p-values	ISS ≥16 p-values	R-square (all model variables)
Hospital mortality	0.0002	0.0010	0.8242	0.22
ICU days	<0.0001	<0.0001	0.5517	0.55
Hospital days	<0.0001	<0.0001	0.2359	0.42
Adverse outcome	0.0102	<0.0001	0.2011	0.31
No commands discharge	0.0459	0.0002	0.9500	0.54
No commands three months	<0.0001	0.0006	0.4157	0.22

## Discussion

Traits and outcomes

The traits of the study group were geriatric, with a mean age of 65, and critically injured, as evidenced by half the patients having an ISS of 16 or greater. Half the study group had ICH AIS of 4-5, which indicates that our head injury population is severely injured or critical. The demographics highlight an older blunt trauma cohort, where almost half the patients had a fall mechanism. We also note that the mortality, no commands at discharge, and no commands at three months rates are less than 10.1%. The mortality rate was low at 6.7%. Out of the 29 deaths, three went to hospice. Combining hospice deaths with hospital deaths is supported in the literature [19]. The R-square values are all >0.22, which implies the regression models are stable. R-square values suggest that we can explain 22% of the outcome with the independently significant variable. Gupta et al. published a review that indicates R-square > 0.15 is meaningful for clinical research, including TBI [20]. Therefore, the low mortality rate does not affect the overall results.

EC-ISS significance

EC-ISS and ICH AIS interactions appear to capture EC injury severity better than ISS ≥16 and may be a better prognostic indicator of outcomes. By treating EC-ISS as a calculated value that quantifies EC injury burden, we showed EC-ISS and ICH AIS to be independent predictors of each of the six undesirable outcomes. ISS ≥16 showed no independent association with any of the six undesirable outcome statistical models. Because ICH AIS and EC-ISS were subcomponent elements of ISS, ISS had a colinear statistical relationship with each. Collinearity was managed by the multivariate regression analyses, which showed that ICH, AIS, and EC-ISS each had independent associations with each of the six undesirable outcome models, but ISS did not. These observations indicate that the fractionated ISS components, ICH AIS and EC-ISS, were better representations for anatomic severity in ICH patients than was ISS.

Although ISS ≥16 is a measure of a severely injured trauma patient, our results add to the literature demonstrating that ISS is imprecise in predicting undesirable outcomes [7].

Skrifvar’s study is the only other study that computed EC-ISS values in patients with TBI. In his study that focused on administration of erythropoietin versus placebo [16], he calculated an EC-ISS using a different methodology, and the median value in his publication was 6. A review of his study shows that EC-ISS >6 occurred in 301 patients and EC-ISS ≤6 occurred in 302. His cutoff values seem somewhat arbitrary. Supplemental Data Content linked to the manuscript showed that EC-ISS >6 and EC-ISS ≤6 patients had similar (P > 0.05) ICU, hospital, and six-month survival and six-month Glasgow Outcome Scale - Extended good outcomes [16]. He showed longer (P <0.001) ICU and hospital LOS [16]. Skrifvars' methodology contributed to results that differed from our findings. Our EC-ISS formula, as well as Skrifvar’s, has not been externally validated and is exploratory. These two formulas are the only ones described in the literature. Our model appears to be more discriminating.

Frequency of independent risk conditions

Increased ICH AIS and increased EC-ISS were independent risk conditions for all six of the undesirable outcomes. The six undesirable outcome models had an r-squared range of 0.22-0.55, suggesting our results are meaningful. 

Decreased GCS was an independent risk condition for four of the six undesirable outcomes (increased ICU stay, increased hospital stay, AO, and not following commands at hospital discharge). An ICH mass effect or increased ICH CT score was an independent predictor for five of the six undesirable outcomes (hospital mortality, increased ICU stay, increased hospital stay, AO, and not following commands at hospital discharge). Increased age was an independent predictor for two of the six undesirable outcomes (hospital mortality and not following commands at three months). Systolic blood pressure <100 mmHg was an independent predictor for three of six undesirable outcomes (increased ICU stay, AO, and not following commands at three months). These risk conditions noted in our data have been well studied and are in alignment with a recent meta-analysis on risk factors for TBI [21].

Higher admission glucose levels were independently associated with three of six undesirable outcome statistical models (increased ICU days, increased hospital stay, and AO). This finding has been supported by other investigators, who have shown postinjury hyperglycemia to be associated with undesirable outcomes [12]. Researchers have provided evidence that postinjury hyperglycemia is related to sympathoadrenal stress response, preinjury diabetes mellitus, or inflammation [13]. Our findings add to the evidence that postinjury hyperglycemia is a risk condition for worse outcomes. The finding that most hyperglycemic patients in the current study were non-diabetic suggests that elevated glucose was secondary to an inflammatory state.

EC injury has been hypothesized to worsen mortality and functional outcomes in TBI secondary to neuroinflammation by increasing peripheral and central neurological responses [22]. Large databases have highlighted EC injury as contributing to worse TBI outcomes without quantifying the degree of EC injury [14]. Multiple animal studies have shown EC injury to be associated with secondary inflammatory responses that exacerbate head injury; however, human models are lacking [21]. Therefore, our research is an important step in quantifying EC injury in ICH patients, and future prospective studies need to be conducted to validate our results. 

Limitations

The current study is retrospective and single-center in design. The data from our level I trauma center in northeast Ohio with a geriatric population may limit applicability to younger trauma populations. The data used in this analysis included the COVID-19 pandemic and did not include entire calendar years. Partial years were included because the data set was derived from a prior publication [17] that focused on the lockdown period in Ohio and the prior two years as a historical period. Partial years, single-center, and geriatric data may limit the applicability and introduce selection bias. Comparing the January 21 to July 21 patients to the July 22 to January 20 patients for each of the three study years would be an entirely different study. Unmeasured confounding factors such as prehospital care, antithrombotic use, comorbidities, and systemic injuries are not accounted for. Future multicenter studies need to be done in order to further test the objectives.

## Conclusions

EC-ISS and ICH AIS outperform ISS in all six undesirable outcome models. The failure of ISS to be an independent predictor for any of the six undesirable outcomes suggests that ICH AIS and increased EC-ISS may have greater importance in geriatric blunt trauma ICH patients. More studies are needed, but EC-ISS could be used in future prognostic outcome modeling of ICH patients. Other ICH patient risk conditions that should be considered as important predictors include increased age, ICH mass effect or increased ICH CT score, hyperglycemia, and systemic admission hypotension.
